# Craig Microscope

**Published:** 1869-08-15

**Authors:** 


### *Craig Microscope.*

Can any body tell us what has become of the individual who advertised the Craig Microscope? We have a letter from one of our subscribers, who somewhat anxiously wishes to know his whereabouts. A publication of our correspondent's letter may be needed. Can the microscope man see it?

